# Predicting ^131^I-avidity of metastases from differentiated thyroid cancer using ^18^F-FDG PET/CT in postoperative patients with elevated thyroglobulin

**DOI:** 10.1038/s41598-018-22656-4

**Published:** 2018-03-12

**Authors:** Min Liu, Lingxiao Cheng, Yuchen Jin, Maomei Ruan, Shiwei Sheng, Libo Chen

**Affiliations:** 1 Department of Nuclear Medicine, Shanghai Jiao Tong University Affiliated Sixth People’s Hospital, Shanghai, 200233 People’s Republic of China; 2 Department of Nuclear Medicine, Shanghai Chest Hospital, Shanghai Jiao Tong University, 200030 Shanghai, People’s Republic of China

## Abstract

The quantitative relationship between iodine and glucose metabolism in metastases from differentiated thyroid cancer (DTC) remains unknown. Aim of the prospective study was to establish the value of ^18^F-FDG PET/CT in predicting ^131^I-avidity of metastases from DTC before the first radioiodine therapy. A total of 121 postoperative DTC patients with elevated stimulated serum thyroglobulin (ssTg) who underwent ^131^I adjuvant therapy or therapy after ^18^F-FDG PET/CT scan were enrolled. The Receiver operating characteristic curve was established to create an optimal cut-off point and evaluate the value of SUVmax for predicting ^131^I-avidity. In our study, the median SUVmax in ^131^I-nonavid metastatic target lesions was also significantly higher than that in ^131^I-avid metastatic target lesions (5.37 vs. 3.30; *P* = 0.000). At a cut-off value of 4.0 in SUVmax, the area under curve was 0.62 with the sensitivity, specificity, positive predictive value and negative predictive value of 75.3%, 56.7%, 76.1%, and 54.8%, respectively. These results suggest that ^18^F-FDG PET/CT may be of great value in identifying metastases in postoperative DTC patients with elevated ssTg before ^131^I administration, leading to an improved management of disease. ^18^F-FDG positive metastatic DTC with SUVmax of greater than 4.0 possesses higher probability of non-avidity to radioiodine.

## Introduction

As the most common endocrine malignancy, the prevalence of thyroid cancer has increased dramatically in the past few decades^1^. In general, differentiated thyroid carcinoma (DTC) are indolent tumors associated with a favorable prognosis, especially in patients with local disease. Resection with or without remnant ablation using radioactive iodine (^131^I, a theranostic agent) remains their mainstay status. However, in the long-term management, especially in patients with advanced cancer, therapeutic strategies should be more cautiously refined^2^. A comprehensive flowchart of the management of advanced DTC including local recurrence/persistence and metastases has been suggested by our group recently^3^.

^131^I is the most important diagnostic and therapeutic agents for metastatic DTC patients^4^. Intense iodine avidity in metastatic lesions usually predicts a favorable outcome. However, up to 10% of metastases become radioiodine-refractory and can not benefit from ^131^I therapy with increased risk of adverse effects^5^. Hence, ^131^I therapy should be confined to selected DTC patients with ^131^I-avid metastases^6^.

Commonly, the primary means to recognize iodine-avid metastases relies on combining the results of post-therapeutic ^131^I whole-body scan (Rx-WBS) and serum thyroglobulin (Tg) test, leading to ineffective treatment inevitable. So, markers predicting the status of ^131^I uptake by metastatic lesions are desirable for timely changing therapeutic regimen. ^131^I diagnostic whole body scan (Dx-WBS) may be traditionally able to detect iodine-avid metastases before ^131^I therapy allowing a more appropriate selection of therapeutic ^131^I activity. However, arguments on the value of post-operative Dx-WBS (with or without SPECT/CT) guiding ^131^I therapy still exist according to 2015 guidelines of American Thyroid Association (ATA)^2^. Moreover, stunning in molecular level and the lack of definitive evidence to avid stunning even using very low dose of ^131^I according to strict imaging protocols have also been reported^7–9^.

Recently, as a non-invasive whole-body imaging technique, the value of ^18^F-FDG PET/CT in DTC has been confirmed by several studies. ^18^F-FDG PET/CT is especially sensitive and effective in detecting metastatic DTC lesions, especially in ^131^I WBS–negative, Tg-positive patients after ^131^I administration^10–14^. Besides, ^18^F-FDG PET/CT has also been demonstrated to aid stratify DTC patients^15–17^. But the experience on the application of ^18^F-FDG PET/CT before ^131^I administration in the identification of metastases in postoperative DTC patients is very limited^17,18^. Feine *et al*. demonstrated that radioiodine-avid thyroid cancer lesions usually be ^18^F-FDG-nonavid, and vice versa^19^. However, metastatic DTC lesions with both uptake of ^18^F-FDG and ^131^I have also been found by previous studies^20–22^. Therefore, the relationship between radioiodine accumulation and ^18^F-FDG metabolism in DTC have not been quantitatively assessed to date. And the value of maximum standardized uptake value (SUVmax) measured on ^18^F-FDG PET/CT in predicting the status of ^131^I uptake in metastases from DTC remains unknown.

Here, therefore, we carried out this dedicated prospective study to evaluate the role of ^18^F-FDG PET/CT in identifying metastatic DTC in postoperative patients with elevated stimulated serum Tg (ssTg) before ^131^I administration. The value of ^18^F-FDG PET/CT in predicting the ^131^I-avidity of metastatic DTC are qualitatively and quantitatively assessed as well.

## Results

### Characteristics of patients

**Figure 1:**
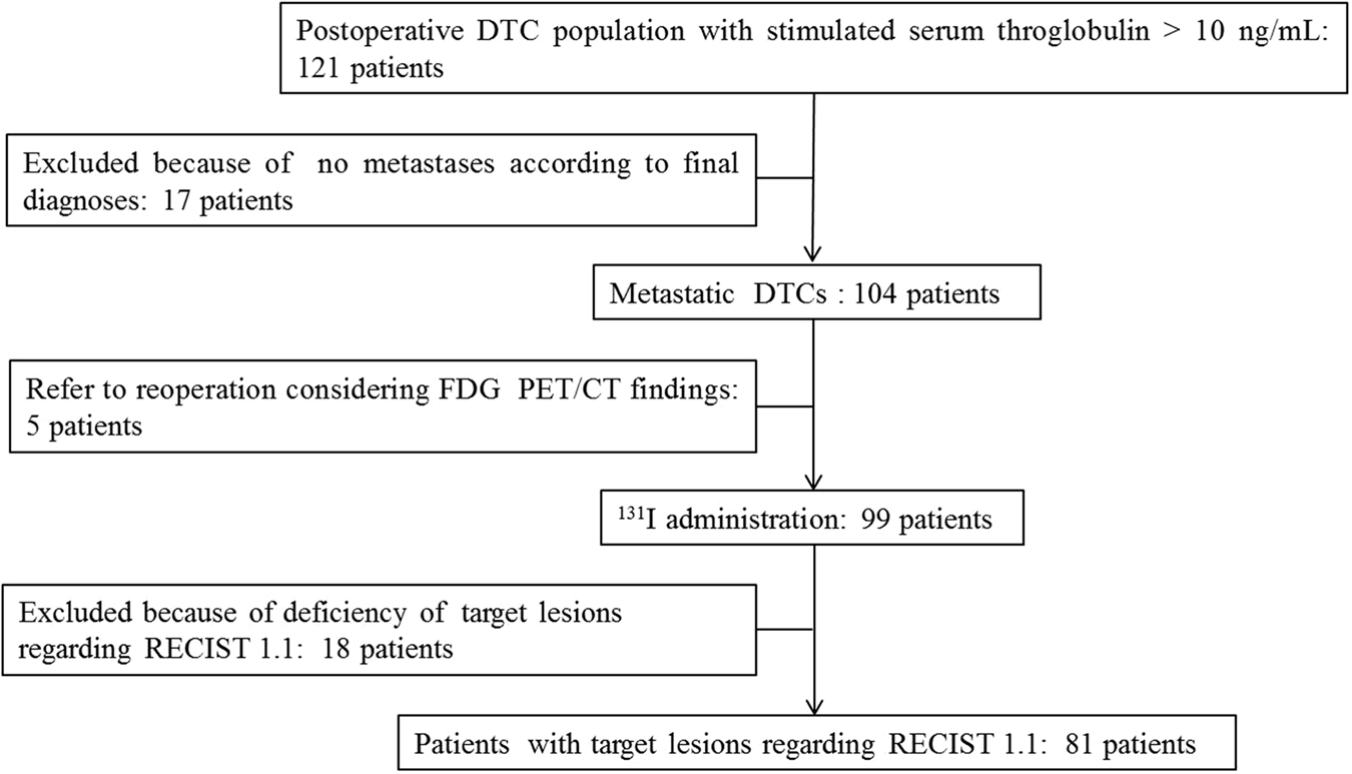
Patient flow diagram of the evaluation of the role of ^18^F-FDG PET/CT in identifying metastatic differentiated thyroid cancers in postoperative patients with elevated stimulated serum thyroglobulin before ^131^I administration.

One hundred and twenty-one consecutive DTC patients (15 with follicular and 106 with papillary carcinoma) constituted the study group including 47 (38.84%) males and 74 (61.16%) females. Eighty-five (70.25%) patients had undergone a total thyroidectomy and 36 (29.75%) patients had undergone a near total thyroidectomy before the enrollment. Mean age of patient at diagnosis was 45.3 ± 12.9 years. The mean interval between thyroidectomy and ^131^I administration was 3.2 ± 1.4 months. Before ^131^I administration, mean TSH and ssTg were 75.6 ± 32.7 mIU/L and 59.6 ± 49.2 ng/mL, respectively. Positive TgAb (>115 kIU/L) was found in nine patients. The median follow-up of all patients was 22.3 months (range, 10.7–34.5 months). A flow diagram is given in Fig. 1.

### Efficacy of ^18^F-FDG PET/CT in identifying metastases in postoperative DTC patients with elevated ssTg

Final diagnostic criteria for metastases from DTC were based on pathological findings, serum Tg levels and other imaging techniques including CT, high-resolution ultrasonography, magnetic resonance imaging, and by correlation with clinical follow-up^23^.

In all the enrolled 121 postoperative DTC patients with elevated ssTg, final diagnosis of metastatic DTC was established in 104 (85.95%) patients. Seventeen (14.05%) subjects were excluded by negative imaging (ultrasonography and CT) and clinical follow up, including three patients (5 lesions located in lymph node) showing false-positive results in ^18^F-FDG PET/CT. Of the 104 DTC patients with metastatic lesions, 92 (88.46%) cases were detected by ^18^F-FDG PET/CT, and the remaining 12 (11.54%) patients showed negative ^18^F-FDG PET/CT results. The sensitivity, specificity, negative predictive value, positive predictive value, and accuracy of ^18^F-FDG PET/CT in the identification of metastases in postoperative DTC patients with elevated ssTg were 88.46% (92/104), 82.35% (14/17), 53.85% (14/26), 96.84% (92/95) and 87.60% (106/121), respectively.

Table 1 summarizes the detailed data of all the 104 patients with postoperative metastatic DTC. The baseline ssTg just before ^18^F-FDG PET/CT scan in patients with ^18^F-FDG-avid metastases and patients with ^18^F-FDG-nonavid metastases were 66.94 ± 56.82 ng/mL and 78.27 ± 48.39 ng/mL (t = 0.95, *P* = 0.34), respectively. Sixty-eight (65.38%) of the 104 patients were at TNM stages of III-IV. No statistically significant differences in the serum TSH level, blood glucose level and uptake time of ^18^F-FDG between the patients with ^18^F-FDG-avid metastases and ^18^F-FDG-nonavid patients were found.

**Table 1:** The demographic and baseline characteristics of patients with postoperative metastatic differentiated thyroid cancer (N = 104).

	Patients with ^18^F-FDG-avid Metastases (n = 92)	Patients with ^18^F-FDG-nonavid Metastases (n = 12)	t/χ^2^	*P*
**Age (Years)**				
Mean ± SD	46.28 ± 13.04	42.41 ± 12.37	1.40	0.17
**Sex**				
Female	55	5	1.43	0.23
Male	37	7		
**Thyroid Cancer Subtype**				
Papillary	76	7	3.20	0.07
Follicular	16	5		
**TNM stage**				
I	16 (13.8%)	2	1.07	0.80
II	14 (10.3%)	3		
III	38 (49.1%)	4		
IV	24 (26.7%)	3		
**Operation Style**				
Total	64	4	1.25	0.25
Near-total	28	8		
**Anti-Tg Antibodies (KIU/L)**				
Mean ± SD	82.95 ± 107.33	75.3 ± 100.92	−1.04	0.30
**Stimulated Thyroglobulin (ng/mL)**				
Mean ± SD	66.94 ± 56.82	78.27 ± 48.39	0.95	0.34
**TSH (mIU/L)**				
Mean ± SD	85.27 ± 42.14	90.50 ± 34.12	−0.67	0.50
**Blood Glucose Level (mmol/L)**				
Mean ± SD	7.82 ± 2.7	7.45 ± 3.6	1.35	0.18
**Uptake Time of** ^**18**^ **F-FDG (minutes)**				
Mean ± SD	58.9 ± 3.54	57.5 ± 4.22	−0.67	0.50

The information obtained from ^18^F-FDG PET/CT scan led to changes in management decision in 39 (32.23%) of the 121 patients. In detail, ^18^F-FDG PET/CT showed residual cervical nodal metastases avid for ^18^F-FDG with the smallest dimension ≥1 cm in 5 patients, who were then referred back to surgery for reoperative neck dissection prior to ^131^I therapy; Besides, an activity of 5.55GBq was enhanced to 7.4 GBq in 34 patients in consideration with their lung and/or bone metastases determined by ^18^F-FDG PET/CT just before ^131^I administration.

### Comparison of findings in metastatic DTC patients between ^18^F-FDG PET/CT and ^131^I Rx-WBS

After excluding 5 patients referred back to surgery for reoperative neck dissection, 99 postoperative metastatic DTC patients were included in the comparison of findings between ^18^F-FDG PET/CT and ^131^I Rx-WBS. Fifty-one (51.52%) patients were concordantly diagnosed using ^131^I Rx-WBS and ^18^F-FDG-PET/CT. Metastatic lesions of 36 patients (36.36%) were not detected by ^131^I Rx-WBS but were found by ^18^F-FDG-PET/CT. Eight (8.08%) patients showed negative ^18^F-FDG PET/CT but positive ^131^I uptake in the chest on ^131^I Rx-WBS. Both ^18^F-FDG PET/CT and ^131^I Rx-WBS showed negative results in the remaining 4 patients (4.04%). Fifty-nine patients showing positive ^131^I uptake on the initial ^131^I Rx-WBS underwent another radioiodine therapy, in which 15 patients showed negative^131^I uptake on the second ^131^I Rx-WBS and 44 patients showed positive findings. In postoperative metastatic DTC patients unfit for reoperation, the sensitivity of ^18^F-FDG-PET/CT (87.88%, 87/99) in the identification of metastatic DTC was significantly higher than that of ^131^I Rx-WBS (59.60%, 59/99) (χ^2^ = 20.45; *P* = 0.000).

### Analyses of factors potentially relative to ^131^I uptake

In patient-based analyses, the clinical characteristics, including age (≤45 years or >45 years), gender (male or female), pathological type (PTC or FTC), qualitative FDG uptake (positive or negative), ssTg (10–100 ng/mL, 100–1000 ng/mL or >1000 ng/mL), TSH (30–60 mIU/L, 60–90 mIU/L, >90 mIU/L), and TgAb (≤115 kIU/L or >115 kIU/L) were analyzed as independent variables using χ^2^ test. After univariate analysis between the groups with ^131^I-avid and ^131^I-nonavid metastases, significant factors related to the ability of ^131^I uptake were age and TSH. After multivariate logistic regression analysis, only TSH remained significant for ^131^I uptake (Table 2).

**Table 2:** Patient-based univariate and multivariate logistic regression analyses of factors potentially related to ^131^I uptake.

Factors	Univariate Analysis		Multivariate Logistic Regression	
	χ^2^	*P* Value	Odds Ratio	*P* Value
Gender (Male vs. Female)	0.723	0.395	/	/
Age (<45 yr vs. >45 yr)	5.703	0.017	−0.626	0.124
Pathological Type (PTC vs. FTC)	0.028	0.866	/	/
Ss-Tg (<100 ng/mL vs. >100 ng/mL)	5.753	0.056	/	/
TgAb (<115 kIU/L vs. >115 kIU/L)	0.552	0.457	/	/
TSH (30–60 mIU/L, 60–90 mIU/L, >90 mIU/L)	12.05	0.002	0.678	0.004
^18^F-FDG Uptake (Positive vs. Negative)	0.945	0.331	/	/

**Figure 2:**
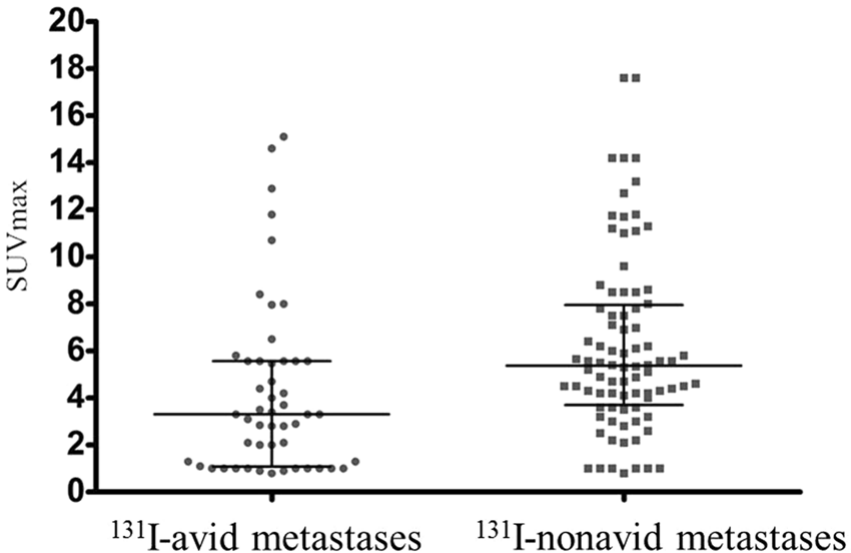
Comparison of SUVmax for ^131^I-avid and ^131^I-nonavid metastatic DTC lesions on ^18^F-FDG PET/CT. *P* = 0.000.

In lesion-based analyses, 18 of 99 patients with metastases which did not meet the criteria of target lesion (RECIST 1.1) were excluded. Then, the size, site, qualitative (positive or negative) and quantitative data (SUVmax) of ^18^F-FDG uptake of 160 target lesions in the remaining 81 patients were taken into account, with details in Table 3. No significant difference in the median size between the ^131^I-avid metastases and ^131^I-nonavid metastases was found (1.12 cm vs. 1.40 cm; *P* = 0.15). There was no significant difference in the sites of metastases between the two groups using the Chi-Square Test (Fisher’s Exact Test) (χ^2^ = 0.33; P = 0.85). However, the percentage of positive ^18^F-FDG uptake in ^131^I-nonavid metastases was 93.5% (87/93), which was significantly higher than that (76.1%, 51/67) in ^131^I-avid metastases group (χ^2^ = 9.98; *P* = 0.002). Further, the median SUVmax was also significantly higher in ^131^I-nonavid metastases than in ^131^I-avid metastases (5.37 vs. 3.33; *P* = 0.000) using Wilcoxon signed-rank sum tests (Fig. 2).

**Table 3:** Analyses of metastatic target lesions of differentiated thyroid cancer according to the avidity for ^131^I.

	^131^I-avid Metastases	^131^I-nonavid Metastases	χ^2^/Z	*P*
Total no.	67	93		
Location			0.33	0.85
Lymph Node	39	54		
Lung	16	26		
Neck	4	6		
Bone	8	7		
Size (cm)			−1.4	0.15
Median	1.12	1.40		
Interquartile Range	1.07	1.23		
^18^F-FDG uptake			9.98	0.002
Positive	51	87		
Negative	16	6		
SUVmax of Target Lesion			−2.6	0.000
Median	3.30	5.37		
Interquartile Range	4.50	4.25

### ROC analyses of SUVmax for predicting ^131^I-avidity

**Figure 3:**
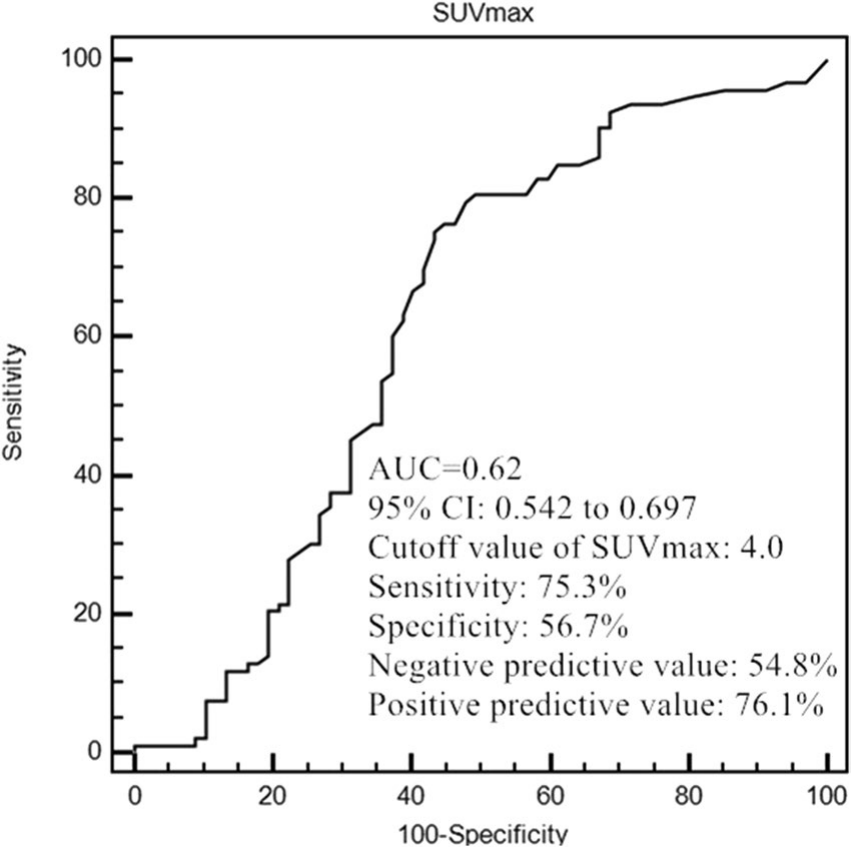
Receiver operating characteristic curve of SUVmax for the prediction of ^131^I-uptake capacity in DTC metastases.

**Figure 4:**
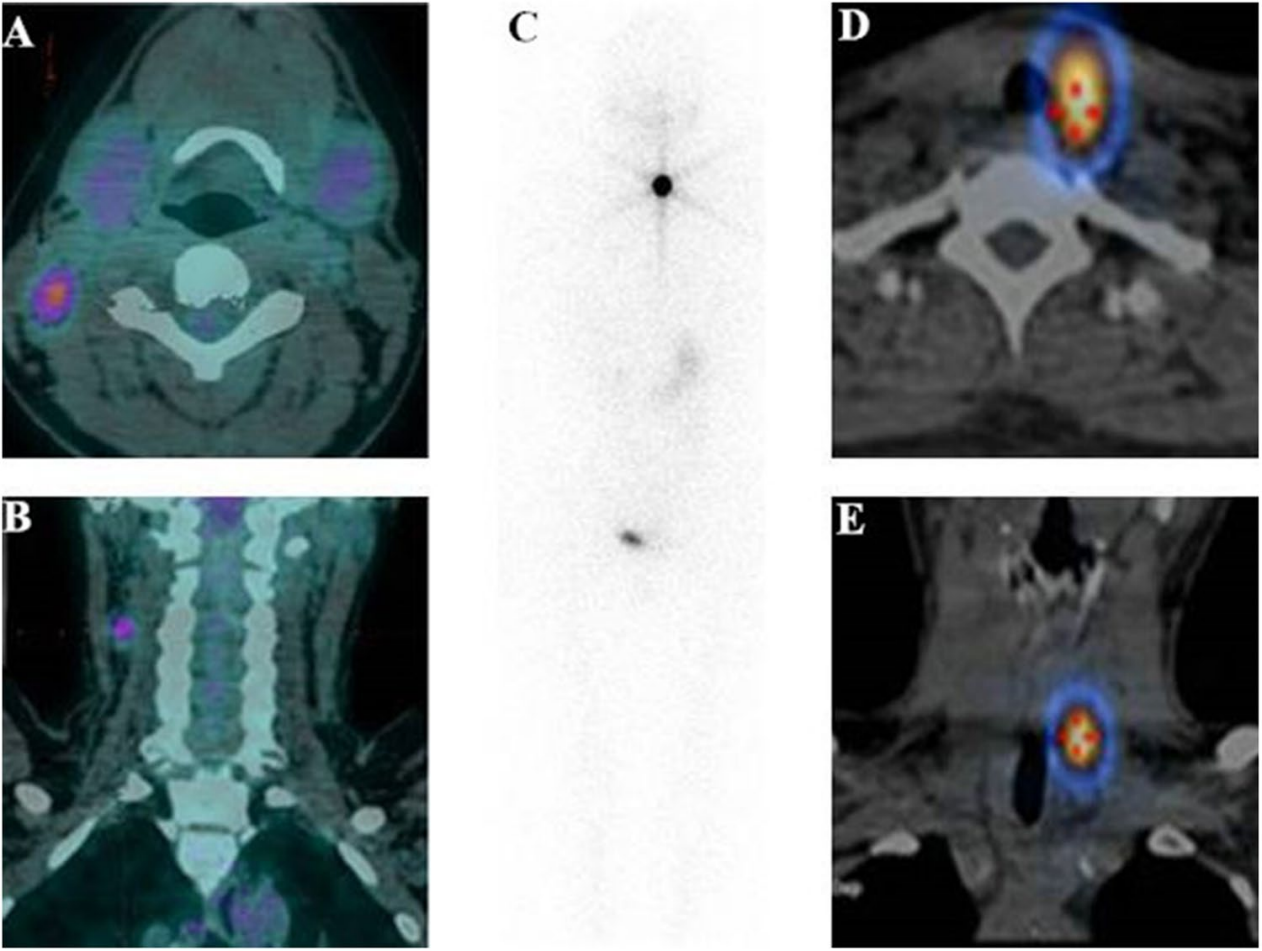
^18^F-FDG-avid papillary thyroid carcinoma (PTC) metastatic lymph nodes non-avid for ^131^I. A 32-y-old female with PTC presented with suspicious metastatic disease 2 months after total thyroidectomy with elevated stimulated serum throglobulin (ssTg) of 78 ng/mL and thyroid-stimulating hormone (TSH) of 102.4 mU/L. Transaxial (**A**) and coronal (**B**) fusion images of ^18^F-FDG PET/CT before the administration of 5.55 MBq (150 mCi) of ^131^I showed obviously radiotracer-avid lymph nodes (LN) (SUVmax = 4.6) in the neck. The ^131^I whole body scan (**C**) and transaxial (**D**) and coronal (**E**) fusion images of ^131^I SPECT/CT of the neck revealed no ^131^I accumulation in the lymph node (not shown) but ^131^I uptake in the thyroid remnant.

**Figure 5:**
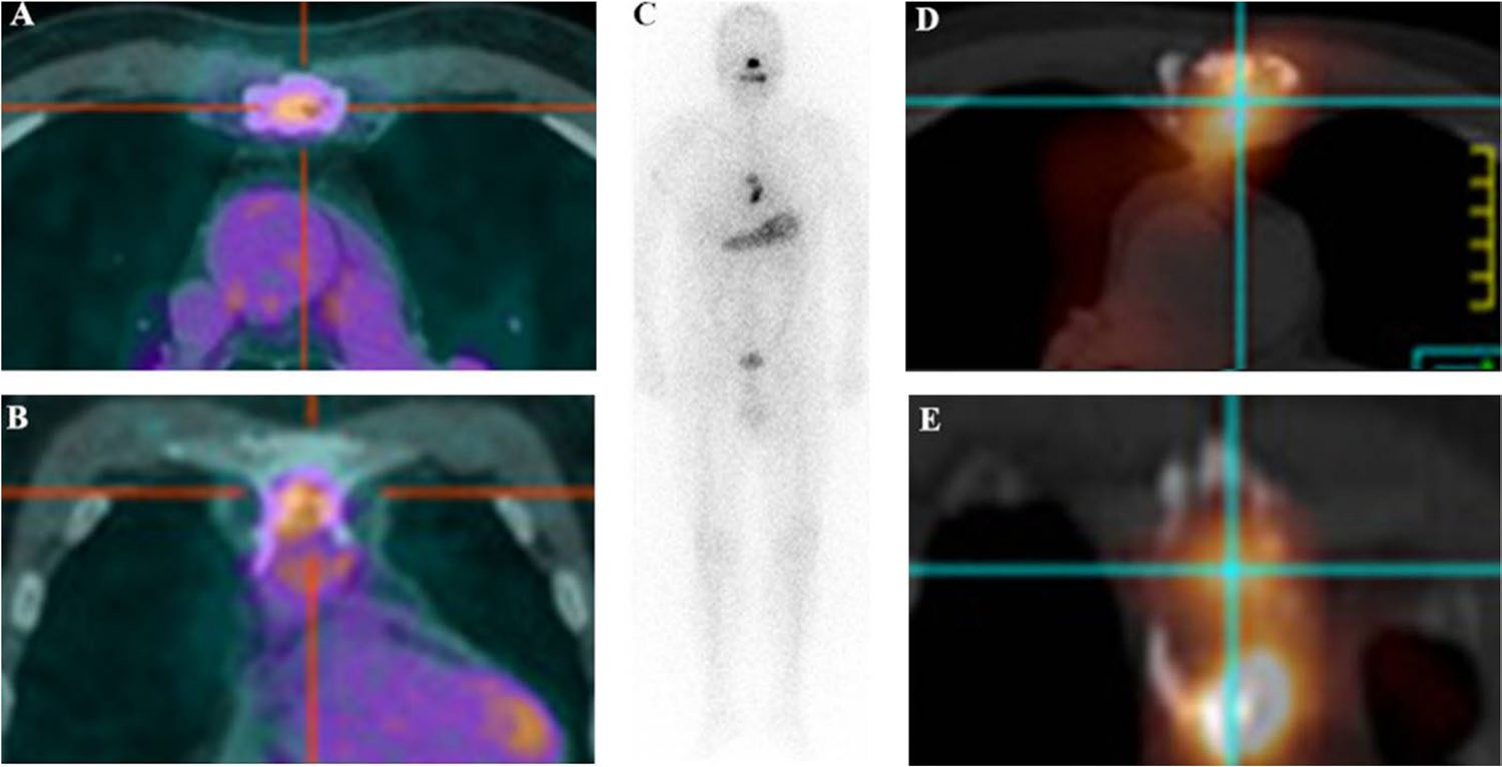
^18^F-FDG-avid papillary thyroid carcinoma (PTC) metastatic sternum lesions avid for ^131^I. A 55-y-old male patient with PTC presented with suspected metastatic disease one month after total thyroidectomy with ssTg of 604.7 ng/mL and TSH of 60.2 mU/L. Transaxial (**A**) and coronal (**B**) image of ^18^F-FDG PET/CT before ^131^I therapy showed sternum lesions with increased ^18^F-FDG uptake (SUVmax = 3.6). ^131^I planar image (**C**) and SPECT/CT (**D**,**F**) after the initial administration of 7.4 MBq (200 mCi) ^131^I correspondingly showed the sternum metastases with increased ^131^I accumulation. Six months later, ssTg decreased to 125.9 ng/mL under TSH stimulation by levothyroxin withdrawl just before the second course of ^131^I administration.

In further ROC analyses, a cut-off value of SUVmax at 4.0 was obtained for predicting the ^131^I uptake status in metastases, with corresponding specificity of 56.7%, sensitivity of 75.3%, and AUC of 0.62. Negative predictive value (NPV) was 54.8% and positive predictive value (PPV) was 76.1% (Fig. 3). In the total of 160 target lesions, 67 (41.9%) lesions showed ^18^F-FDG uptake with SUVmax ≥4.0 (Median: 2.3; interquartile range: 2.0), containing 16 (23.9%) ^131^I-avid metastases and 51 (76.1%) ^131^I-nonavid metastases demonstrated by the subsequent ^131^I Rx-WBS (Fig. 4). Ninety-three target lesions showed ^18^F-FDG uptake with SUVmax <4.0 (Median: 7.1; interquartile range: 6.6), including 51 (54.8%) ^131^I-avid metastases and 42 (45.2%) ^131^I-nonavid metastases (Fig. 5).

## Discussion

In the current prospective study, we initiated ^18^F-FDG PET/CT scans just before ^131^I administration in postoperative DTC patients with elevated ssTg, which was compared directly with the subsequent ^131^I Rx-WBS. We observed significantly higher possibility of ^18^F-FDG uptake in ^131^I-nonavid DTC metastases than in ^131^I-avid ones (95.3% vs. 76.1%), which is generally in accordance with the previously published data on ^18^F-FDG PET/CT scans after ^131^I administration^17,24^. Lesion-based analyses demonstrated that the percentage of positive ^18^F-FDG uptake and the median SUVmax in ^131^I-nonavid metastases were significantly higher than those in ^131^I-avid metastases.

In the process of dedifferentiation of DTC, up-regulated glucose metabolism combined with down-regulated iodine metabolism coexist^25–27^. We have even shown down-regulated glucose metabolism combined with up-regulated iodine metabolism in papillary thyroid carcinoma cells treated with tyrosin kinase inhibitors by an recent *in vitro* study, which elucidated partially the underlying mechanism of such “flip-flop” phenomenon and lay a foundation of the present study^28^. Theoretically, the level of glucose metabolism can be both qualitatively and quantitatively assessed by ^18^F-FDG PET/CT. It is reasonable to establish a possible SUVmax cut-off point over which ^131^I-nonavid metastases from DTC should be suspected. According to the cut-off value of 4.0, if SUVmax of metastases were higher than 4.0, a ^131^I-nonavid metastatic DTC lesion should be suspected and adjuvant therapy for enhancing or restoring the ability of trapping iodine during ^131^I treatment might be indicated. Meanwhile, according to the positive predictive value as 76.1%, ^18^F-FDG positive metastatic DTC with SUVmax of greater than 4.0 possesses higher probability of non-avidity to radioiodine. Further studies aiming at enhancing the value of ^18^F-FDG PET/CT in this aspect are still needed.

Interestingly, in patient-based analysis (Table 2), however, the status of ^18^F-FDG uptake in ^131^I-avid group was not significantly different from that in ^131^I-nonavid group, indicating that only per-patient qualitative analysis of ^18^F-FDG PET/CT is not sufficient to distinguish ^131^I-nonavid metastases from those avid to ^131^I. The status of ^18^F-FDG uptake in different metastatic lesions could be different even in an individual, which can be explained by the multicentricity and polyclone of DTC^29–31^. Our findings revealed that lesion-based analyses and quantitatively assessing the data of ^18^F-FDG PET/CT using SUVmax to predict ^131^I-avidity for metastatic DTC would be more reliable than qualitative per-patient evaluation only. Moreover, in predicting the capability of DTC in accumulating ^131^I, other isotopes (^123^I and ^124^I) and the topography of primary malignant thyroid nodule can be resorted^32–34^. However, difficult availability of such isotopes and the small sample size of enrolled patient with metastatic lesions represent major limitations. And since the value of molecular testing in guiding postoperative ^131^I therapy has not be established well, no recommendation in this aspect can be provided at present^2^.

The level of serum Tg is related to the amount of neoplastic tissue in postoperative patients^35–37^. In those patients with elevated serum Tg (generally >10 ng/mL) and negative ^131^I WBS, ^18^F-FDG PET/CT is complementary to ^131^I WBS for locating lesions according to 2015 ATA guidelines^2^. Notably, in our study, ^18^F-FDG PET/CT identified 76.0% cases with metastatic DTC lesions and changed the management decision in about one-third of the enrolled patients in our study, suggesting an important clinical utility of ^18^F-FDG PET/CT in postoperative patients with elevated ssTg before ^131^I administration. The relatively high diagnostic value of ^18^F-FDG PET/CT in the identification of metastases in postoperative DTC patients may be owing to a relatively high percentage of advanced stages, with stages of III-IV in nearly 56.20% of DTC patients.

In addition, we further investigated the potential patient-based factors related to ^131^I uptake. After multivariate logistic analysis, only TSH remained significant for ^131^I uptake, demonstrating that the TSH level is a significant factor that might influence the ^131^I uptake of DTC metastases. The ^131^I uptake by metastatic DTC might continue to increase as TSH rose to a higher level. Recently, one study has also confirmed that ssTg measured under a higher preablative TSH level might be more convincing as a prognostic marker for DTC^38^. In our study, ^18^F-FDG PET/CT was carried out just one day before ^131^I administration to maximize TSH stimulation without additional preparation and minimize inconvenience to patients.

We admit that there are several limitations in our study. First, in reflecting glucose metabolism, only SUVmax was focused on in the present study, other parameters such like SUV normalized to lean body mass, or body surface area, may be worthy of assessment in the coming studies. Meanwhile, many physiologic and technical variables may affect the outcome of SUVmax, resulting in difficulties of its reproducibility^39,40^. Second, our studies enrolled patients with metastases only in lymph node, lung and bone. So the predictive value of SUVmax in metastatic DTCs in other sites is still unknown. Third, the exact prognostic value, if any, of ^18^F-FDG PET/CT via survival analyses of such indexes remains to be more clearly established.

## Conclusion

Our study suggest that ^18^F-FDG PET/CT can play vital role in identifying metastases in postoperative DTC patients with elevated ssTg (>10 ng/mL) before ^131^I administration, leading to refined management of disease. ^18^F-FDG positive metastatic DTC with SUVmax of greater than 4.0 possesses higher probability of non-avidity to radioiodine.

## Materials and Methods

### Study design and population

We prospectively enrolled postoperative DTC patients who received total or near total thyroidectomy by our general surgery and were suspected to have metastatic disease with elevated ssTg levels (>10 ng/mL). Central neck lymph node removal was conducted in all DTC patients without the history of other malignant tumors. All patients had undergone thyroxin withdrawl for 4 weeks before ^131^I administration. The prescribed ^131^I activity was either 5.5 GBq (150 mCi) for local metastases and suspected but unproven metastases (adjuvant therapy) or 7.4 GBq (200 mCi) for distant metastases. The findings of ^18^F-FDG PET/CT scans and ^131^I Rx-WBS were directly compared.

This study was approved by the ethics board of Shanghai Jiao Tong University Affiliated Sixth People’s Hospital before its initiation. All participants were fully informed of details of the study with the information sheet and signed in the consent form prior to their inclusion in the study. We confirm that all methods were carried out in line with the relevant guidelines and regulations.

### ^18^F-FDG PET/CT scans

All ^18^F-FDG PET/CT images were performed one day before radioiodine administration in our department of nuclear medicine. All patients fasted for at least 6 h and the blood glucose level was less than 150 mg/dL (8.3 mmol/L) before intravenous injection of ^18^F-FDG at a dose of 4.44 MBq/kg (0.12 mCi/kg) body weight. The detailed parameters referred to the article of Jeong Won Lee *et al*.^22^.

### Post-therapeutic ^131^I scans

The post-therapeutic ^131^I scans including Rx-WBS and SPECT/CT (Millennium VG and Hawkeye; GE Healthcare) were used 3 days after an oral therapeutic dose of ^131^I as described previously by our group^23^.

### Criteria of target lesion and final diagnoses

According to RECIST 1.1, we chose up to 5 lesions per patient and up to 2 per organ as target lesions in ^18^F-FDG PET/CT images, and monitored in the subsequent post-therapeutic ^131^I scans^41^. ^18^F-FDG uptake in tumor was quantified as SUV_max_ (the maximum activity concentration of ^18^F-FDG divided by the injected dose and corrected for the body weight of the patient)^40^.

### Statistical analyses

SPSS version 16.0 (SPSS, Inc. Chicago, IL, USA) were used for statistical analyses. The significance of categorical data were compared using Fisher exact tests and Chi-Square Tests. The nonparametric Wilcoxon rank sum test was applied to evaluate quantitative data when it was not normally distributed. The factors related to ^131^I uptake were investigated using logistic regression analysis. Receiver-operating characteristic (ROC) curve analysis was used to obtain the cut-off value of SUVmax for differentiating ^131^I uptake status in metastatic lesions. Sensitivity, specificity, positive predictive value and negative predictive value of the cutoff value of SUVmax were calculated. All *P* values reported were 2-sided, and a *P* value < 0.05 was considered to indicate statistical significant.
